# Exploitation of Selected Sourdough *Saccharomyces cerevisiae* Strains for the Production of a Craft Raspberry Fruit Beer

**DOI:** 10.3390/foods12183354

**Published:** 2023-09-07

**Authors:** Viola Galli, Manuel Venturi, Simona Guerrini, Silvia Mangani, Damiano Barbato, Gianni Vallesi, Lisa Granchi

**Affiliations:** 1 Department of Agriculture, Food, Environment and Forestry (DAGRI), Via San Bonaventura, 13-50145 Florence, Italy; viola.galli@unifi.it (V.G.); gianni.vallesi.1@gmail.com (G.V.); lisa.granchi@unifi.it (L.G.); 2 FoodMicroTeam s.r.l., Academic Spin-Off of the University of Florence, Via Santo Spirito, 14-50125 Florence, Italy; simona@foodmicroteam.it (S.G.); damiano@foodmicroteam.it (D.B.)

**Keywords:** fruit beer, sourdough *Saccharomyces cerevisiae* strains, aromatic profile, raspberry, craft beer, non-brewing yeasts

## Abstract

Recent interest in the special beer category has encouraged the search for novel brewing materials, including new ingredients and novel yeast strains, in order to differentiate the finished products. The aim of this work was to select non-brewing *S. cerevisiae* strains for the production of a fruit beer with raspberry. The in vitro tests and the wort fermentations allowed the selection of two sourdough *S. cerevisiae* strains, showing high maltose and maltotriose consumption, high ethanol production, and high viability. Fruit beers (FB) and control beers (CB) without raspberries were prepared. Fruit addition accelerated sugar consumption (7 days compared to 13 days) and increased ethanol and glycerol production by yeasts. Raspberry addition and the inoculated yeast strongly affected the aroma profile of beers. FB samples showed a higher amount of volatile organic compounds (VOCs); the most represented classes were alcohols, followed by esters and acids. FB inoculated by the selected *S. cerevisiae* SD12 showed the highest VOCs concentration (507.33 mg/L). Results highlighted the possible application of sourdough yeast strains for the brewing process, which, combined with raspberry addition, can be exploited for the production of beers with enhanced aromatic features and suitable chemical properties.

## 1. Introduction

Beer is one of the most popular and consumed alcoholic beverages in the world. Although a huge beer variety, the production styles can be generally grouped into three categories: “Ale” beers, “Lager” beers and a third category, which includes spontaneously fermented or “Lambic” beers. *Saccharomyces cerevisiae* is the leading microorganism for Ale production, while Lager is usually brewed with *S. pastorianus* [1]. In recent years, the growing trend in the craft beer segment has highlighted the need for innovative products aiming to obtain beers with unique, peculiar properties. To achieve this goal, the addition of different ingredients or the choice of microbial strains has been proposed.

Fruit beers are included in the special beer category; they are obtained by adding raw fruits directly, fruit extracts, or fruit-flavoured additives during different phases of the production process, such as fermentation, maturation, or bottle refermentation [2]. The market for fruity beers has increased over the last decade, and it is predicted to reach around 379.5 billion by 2027, whereas in 2021, it was 266.9 billion [3]. Usually, fruit can be added in two stages of the brewing process: during the so-called hot phase (mash or boiling step), where the high temperature prevents microbial contamination but affects fruit flavour, or during the cold phase (first fermentation or cold maturation) where the aromatic characteristics remain unaltered, but microbial spoilage risks are increased. Indeed, as provided by Italian law [4], craft beer must not be stabilised by pasteurization or microfiltration, increasing the risks of spoilage. Beer enrichment with fruits can provide peculiar colour, taste, and flavour to the final product, as well as a higher acidity and can increase the bioactive compounds, depending on the type of fruit. For centuries till now, fruits have been used as beer ingredients in Belgian cherry Lambic (‘Kriek’) or raspberry Lambic (‘Framboise’) beers. A wide variety of fruits has been added to beer, such as persimmon [5], cherry and plum [6], mango [7], apricot [8], goji berries [9], and red raspberry [10]. Red raspberries (*Rubus idaeus* L.) are members of the Rosaceae family; they are widely consumed as fresh, frozen, or in processed forms such as jellies, jams, and juices. They are known for their nutritional quality, containing a variety of beneficial compounds such as essential minerals, vitamins, fatty acids, and dietary fibres and a wide range of polyphenolics, particularly ellagitannins and anthocyanins [11]. Moreover, they are appreciated for their characteristic flavour and aroma, which is a result of nearly 300 volatile compounds, especially those belonging to the monoterpenes and acids class of compounds [12].

Together with raw material, the yeast starter used for both primary fermentation and bottle refermentation is another factor that strongly affects beer flavour [13]. A central role is attributed to the yeast strain due to the strain dependence on several technological properties. In craft beer production, this aspect is of particular importance as the typical and distinctive sensory characteristics are crucial to confer the specificity and overall quality of the final product. In this context, the development of new microbial starters can be oriented towards improving already available yeast strains or by selecting new strains from different fermented matrices. The ability to quickly and completely utilise the available fermentable carbohydrates, ethanol and glycerol production, hop tolerance, and the relative production of aroma compounds of interest are considered requisites in the selection of strains for brewing [14]. Indeed, several studies are focusing on improving microbial biodiversity for beer production, including the possible use in the brewing process of different yeasts, such as *Saccharomyces* strains isolated from other fermented or unfermented foods (e.g., cachaça, ripe fruits, sourdough) [15,16,17] and non-*Saccharomyces* strains [18]. For instance, sourdough yeasts were used as an alternative source of starters for craft beer production by Marongiu et al. [19]. They found a high carbohydrate assimilation rate of sourdough yeasts comparable to or higher than that of the brewer’s strain. Mascia et al. [17] compared two craft beers produced by using a commercial *S. cerevisiae* starter and a strain isolated from sourdough; the two beers showed significant differences in the triangle test, with a tendency to prefer beer brewed with the sourdough yeast. *S. cerevisiae* strains, isolated from the winery and tested as inoculum in the refermentation phase, led to beers with peculiar and distinctive aroma profiles, particularly rich in volatile compounds, responsible for fruity and flowery aromas, compared to the commercial strain [20].

In light of this, the aim of the present study was first to investigate the technological properties of non-commercial strains of *S. cerevisiae* isolated from sourdoughs and wineries. The selected candidate and a commercial brewing starter were used to produce beers with the addition of raspberry puree; the effects of the strains and fruit addition were evaluated by assessing the physicochemical and aromatic features of beers.

## 2. Materials and Methods

### 2.1. Strains and Culture Conditions

Twenty-two *S. cerevisiae* strains, previously isolated and identified, were used in this study. Nineteen strains were isolated from different Italian sourdoughs, three strains from three different Tuscan wineries; they all belonged to the collection of the Department of Agricultural, Food and Forestry Systems of the University of Florence (Italy) and were used in this study (Table 1). In addition, seven commercial yeast strains used for bakery, brewing and wine production were also tested.

Before assaying, they were aerobically cultured for 24 h at 30 °C in YEPD, a medium containing (in g/L) glucose 20, yeast extract 10, peptone 20. For the inoculum in must, cells were recovered by centrifugation (5000× *g* for 20 min), successively washed in physiological solution and re-suspended in the wort.

### 2.2. Microbiological Analysis

Microbial counts of yeasts were performed by serial dilution method. Briefly, 1 mL of samples was homogenised with 9 mL of sterile saline solution. Serial dilutions were made, and the diluted suspensions were plated on WL Nutrient Agar medium (Thermo Fisher Scientific Inc., Waltham, MA, USA). Plates were incubated for 48 h at 30 °C in aerobic conditions.

### 2.3. Screening of S. cerevisiae Strains for Technological Traits

Technological characteristics, including growth with maltose and maltotriose as a unique carbon source at two temperatures, resistance to two percentages of ethanol, and production of hydrogen sulphide (H_2_S), were investigated in order to select strains with better performances for beer production.

#### 2.3.1. Growth of Different Carbon Sources

Yeast cultures, grown 24 h, were inoculated 1% *v*/*v* in YEPD with maltose (YEPD-M) or maltotriose (YEPD-MT) as carbon sources instead of glucose. Yeast growth was determined by measuring optical absorbance at 660 nm using a spectrophotometer (V730, Jasco Inc., Tokyo, Japan) at the beginning and after 6 and 24 h at 12 °C and 20 °C.

#### 2.3.2. Ethanol Tolerance

For ethanol tolerance assay, dilutions of exponential pure cultures were spotted onto Petri dishes containing MEA (malt extract agar) solid medium (malt extract 30 g/L, peptone 5 g/L) added with ethanol at 5 and 10% (*v*/*v*). Plates were incubated at 20 °C and observed after 24 h and 48 h. Growth was estimated by using a four-level scale: 0 = no growth, 1 = weak growth, 2 = growth, and 3 = intense growth, according to Matraxia et al. [21]. Yeast strains were also inoculated 1% *v*/*v* in YEPD-M liquid medium added with 5% or 10% (*v*/*v*) of ethanol. Yeast growth was determined by measuring optical absorbance at 660 nm using a spectrophotometer (Jasco Inc.) at the beginning and after 6 and 24 h at 20 °C.

#### 2.3.3. Hydrogen Sulphide Production

To evaluate the production of hydrogen sulphide (H_2_S), the strains were cultured onto bismuth sulphite agar (Biggy Agar) (Nickerson medium, Merck KGaA, Darmstadt, Germany) according to Jiranek et al. [22]. H_2_S production was estimated by colony blackening after 3 days of incubation at 28 °C, using a five-level scale: 0 = white, 1 = beige, 2 = light brown, 3 = brown, 4 = dark brown, and 5 = black. *S. cerevisiae* US-05 (Fermentis, Lesaffre, France) and *Starmerella bacillaris* were used as negative (0 = white) and positive controls (5 = brown), respectively.

### 2.4. Wort Fermentation Ability of S. cerevisiae Strains

A commercial Coopers DIY Beer Real Ale (Coopers Brewery, Adelaide, Australia) brewing extract was used. Brewing extract was mixed and diluted with water according to the manufacturer’s instructions in order to maintain a temperature of ca 25 °C. Final Wort composition was as follows: total sugar content 51.10 ± 2.0 g/L (maltose 31.20 g/L and maltotriose 9.50 g/L FAN 169.01 ± 3.00 (mg/L), and initial pH of 4.52 ± 0.10. Fermentations were carried out in duplicate for 7 days in 250 mL flasks containing 200 mL of the prepared malt. The selected *S. cerevisiae* strains were singly inoculated at two 2 × 10^6^ cells/mL. Fermentations were carried out in duplicate at 20 °C and flasks were weighed once a day after gentle mixing (1 min) in order to monitor the fermentation progress (CO_2_ evolution), measuring the weight loss of the flasks until the end of the fermentation (constant weight for three consecutive days). For microbiological and chemical analyses, samples were collected on days 1, 2, 3, 6 and 7. Microbiological counts were performed as described in Section 2.2, and chemical analysis as reported in Section 2.6. All analyses were performed in duplicate.

### 2.5. Small-Scale Brewing by Selected S. cerevisiae Strains

Stainless steel tanks (5 L of volume) containing 3 L of beer wort were inoculated by using the three best-performing strains of *S. cerevisiae* at a concentration of ca 2 × 10^6^ cells/mL to obtain fruit beer (FB) with raspberry puree addition, and control beer (CB) without fruit. Wort composition was adjusted in order to increase total sugar content, and the composition was the following: total sugar content 86.25 g/L (maltose 54 g/L and maltotriose 16.85 g/L); FAN 281.01 ± 3.00 (mg/L), and initial pH of 5.15 ± 0.10. After 48 h of fermentation at 20 °C, the raspberry puree (Ravifruit, Anneyron, France) was added to the wort at 15% *w*/*v*. Raspberry puree sugar composition was as follows: glucose 91 g/L, fructose 88 g/L and pH 3.13. All the inoculated worts were left to ferment until sugars were almost depleted (<10 g/L). At the end of the primary fermentation, they were bottled in 66 cL glass bottles for the secondary fermentation. In this step, 5 g/L of sucrose was added to each bottle. Finally, bottles were placed in an incubator at 20 °C for 14 days. Final products were stored at 4 °C; no pasteurisation or filtration was carried out. During the primary fermentation, microbiological and chemical analyses were carried out on samples collected at the beginning, at days 2, 6 and 7 for fruit beer, and beginning days 2, 7, 8, 9, and 13 for control beers. Beers were analysed at the beginning and at the end of the refermentation process. All fermentations were carried out in duplicate.

### 2.6. Chemical and Analytical Determination of Wort and Beers

The pH values were determined by a pH meter (Metrohm Italiana Srl, Varese, Italy). Maltose, maltotriose glucose, fructose, glycerol, and ethanol contents in inoculated worts and beers were determined by high-pressure liquid chromatography (HPLC) according to Guerrini et al. [23]. Maltose, maltotriose, glucose and fructose were often reported as total sugars. Separation was obtained with a Rezex ROA organic acid H+ column (300 × 7.8 mm; Phenomenex, Castel Maggiore, Bologna, Italy), preceded by a security guard cartridge (carbo H 4 × 3.0 mm ID) connected to a refractive index detector (Varian, Prostar 350, Varian Inc, Palo Alto, CA, USA) and UV-VIS detector (λ = 210 nm) (Pro star 335, Varian Inc, Palo Alto, CA, USA). Elution was performed at 65 °C with 0.013 N H_2_SO_4_ eluent at a flow rate of 0.6 mL/min. Ammoniacal nitrogen and α–aminoacidic nitrogen (FAN) were determined by using enzymatic kits (Steroglass S.r.l. Perugia, Italy) following the manufacturer’s instructions. Before the assays, beer worts and beers were diluted ten-folds with distilled water. The total phenolic compounds of the beers were determined according to Kawa-Rygielska et al. [24] with some modifications. 0.5 mL of beer sample and 2.5 mL of Folin–Ciocalteu reagent were pipetted into cuvettes. After 3 min, 2.5 mL of a 20% aqueous solution of sodium carbonate (Na_2_CO_3_) and 2 mL of distilled water were added. The absorbance was measured at the 765-nm wavelength after 1 h of incubation, and the results were expressed as mg of gallic acid equivalents (GAE) per L of beer.

### 2.7. Aromatic Profile Determination of Beers

The aroma profile of fruit and control beers was carried out according to Guerrini et al. [25]. The AAC profile was determined by gas chromatography with mass spectrometry detection (GC-MS) after solid-phase microextraction (SPME) sampling of the headspace of sampling vials in equilibrium with the liquid (HS-SPME-GCMS). Chromatographic separation was performed using an Agilent 7890 gas chromatograph linked to an Agilent 5975 Mass Selective Quadrupole Detector (Agilent Technologies, Inc, Santa Clara, CA, USA) operating in scan mode, using an HP-INNOWAX capillary column (50 m × 0.2 mm i.d., film thickness 0.4 μm). Internal standard (ISTD) (0.05 mL) was added to the sample. Tentative compound identification was carried out by comparing the mass spectra of the separated compounds and their retention indices with those reported in the Nist08 spectral database following dynamic background compensation with Agilent MassHunter Quantitative Analysis (MS).

#### Qualitative and Quantitative Analyses

AACs were identified by comparing the experimental retention time and mass spectra with those obtained from the analysed standard (for quantified compounds) or the experimental Kováts retention indices and mass spectra with those reported in the NIST chemistry webbook (National Institute of Standards and Technology, Mass Spectral Search Program v. 2.0, Washington, DC, USA spectral libraries). Quantitative data were obtained using the internal standard method after normalisation of the peak area of each compound in relation to that of the appropriate internal standard.

### 2.8. Statistical and Data Analysis

The level of statistical significance was determined using one-way ANOVA or two-way ANOVA followed by Tukey’s Test (for multiple groups) (GraphPad Prism 8 software package, San Diego, CA, USA). A *p-value* of <0.05 was considered to be significant. The principal component analysis (PCA) was carried out using STATISTICA 7 software.

## 3. Results and Discussion

### 3.1. Yeast Screening for Technological Characteristics

A screening of several brewing characteristics of the 29 *S. cerevisiae* strains considered in this study was carried out. To select the strains showing better performances, growth with maltose or maltotriose as a unique carbon source, resistance to two percentages of ethanol, and production of hydrogen sulphite (H_2_S) were investigated.

**Figure 1:**
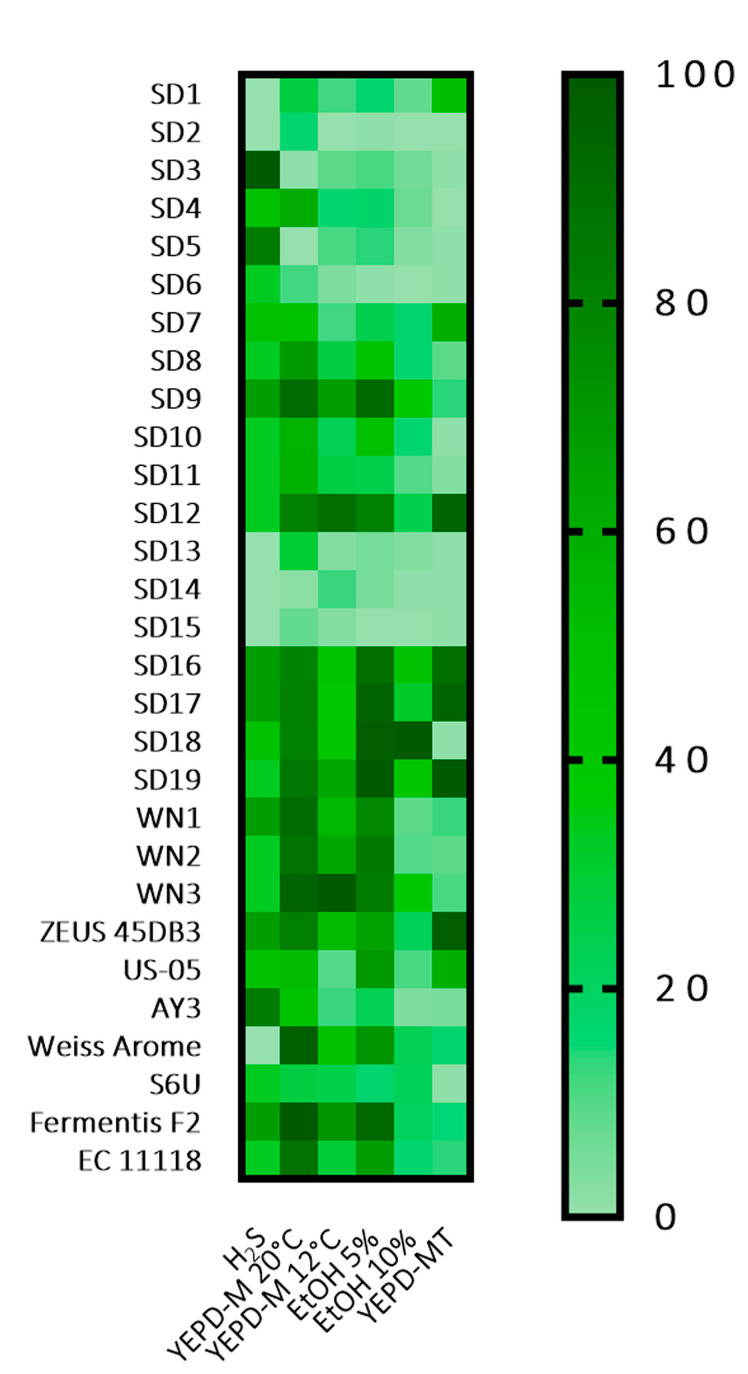
Heatmap based on *S. cerevisiae* strains technological features assessed by the screening: hydrogen sulphide production (H_2_S), growth in YEPD with maltose at 20 °C (YEPD-M 20 °C) and 12 °C (YEPD-M 12 °C); tolerance to 5% ethanol (EtOH 5% *v*/*v*), tolerance to 10% ethanol (EtOH 10% *v*/*v*); growth in YEPD with maltotriose (YEPD-MT). The values of each activity were standardised to range from 0 to 100. Colours correspond to standardised values from the worst performance (light green) to the best performance (dark green).

The obtained data were standardised, and elaborated through a heatmap (Figure 1). Results suggested a high variability among the tested strains that was not related to the source of isolation. As regards sulphite production, a compound responsible for off-flavours, the majority of the strains were characterised by very low production of H_2_S on Biggy agar plates (white—light brown colony); they were considered low H_2_S producers. 5 sourdough *S. cerevisiae* strains and the commercial Weiss Arome strains showed a higher H_2_S production, reaching the score of 3. Maltose and maltotriose assimilation was tested since they represent the most abundant fermentable sugars in brewer’s wort, varying from 50 to 60% and 15 to 20%, respectively (Figure S1). Hence, their utilisation is a fundamental requisite in order to avoid low ethanol yields and the presence of residual sugars imparting sweetness to the final product. Two maltose fermentation temperatures were tested, 20 °C, which is the fermentation temperature for Ale beers, and 12 °C, which is similar to that of Lager beers, by including maltose as a unique carbon source. In general, the absorbance values after 48 h were higher at 20 °C compared to 12 °C, and the median values decreased from 1.13 to 0.19 with a lower fermentation temperature. The sourdough strains generally showed a weak ability to consume maltose; absorbance values above the 75th percentile (greater than 1.41) in YEPD maltose medium at 20 °C were reached by strains SD9, EC1118, AEB Weiss, WN1, WN2, WN3 and ZEUS 45DB3, the highest value by the commercial strain Fermentis F2. Absorbance values above the 75th percentile (greater than 0.352) in YEPD maltose medium at 12 °C were achieved by strains SD9, SD12, SD19, WN1, WN2, WN3 and Fermentis F2. However, in these conditions, the maximum absorbance value (0.64) was exhibited in 48 h by the WN3 strain. In this experiment, the growth with maltose was observed for the strains isolated from wineries, the commercial strains and 13 out of 19 sourdough strains (68%). To assess the strains’ ability to hydrolyse maltotriose, a trisaccharide consisting of three glucose molecules linked with α-1,4 glycosidic bonds, a further investigation on carbohydrate consumption was carried out. Assimilation of maltose and maltotriose requires transport across the cell membrane and an intracellular α-glucosidase to cleave them into glucose molecules. However, maltotriose has the lowest priority for uptake cells [26]. Eight strains revealed the highest maltotriose assimilation capacity, six sourdough strains, the commercial baker’s yeast and the brewer’s yeast US-05. Yeast growth was also evaluated by measuring absorbance, both in YEPD medium with maltose and in the presence of ethanol at two different concentrations at 20 °C: 5% and 10% (*v*/*v*). However, the addition of ethanol inhibited yeast growth to different extents. Indeed, the median value of YEPD with ethanol 5% was 0.514; this value further decreased with the increase in ethanol content, up to 0.12 with 10% of ethanol. The strains revealing better ethanol tolerance were SD9, SD15, SD16, SD18, SD19 WN2, WN3 and Fermentis F2; whereas SD2, SD6, SD15 were unable to grow. SD9, SD16, SD17, SD18, SD19, and WN3 displayed higher values also with 10% of ethanol, together with SD12 and AEB Weiss. 15 out of 29 tested strains did not show any growth. These results were confirmed by the tests carried out on MEA medium.

From the results of the screening, SD9, SD12, SD19 (isolated from sourdoughs) and WN3 (isolated from a winery) strains were selected for wort fermentation tests; in addition, the commercial brewing strain US-05 was chosen as control.

### 3.2. Wort Fermentation

**Figure 2:**
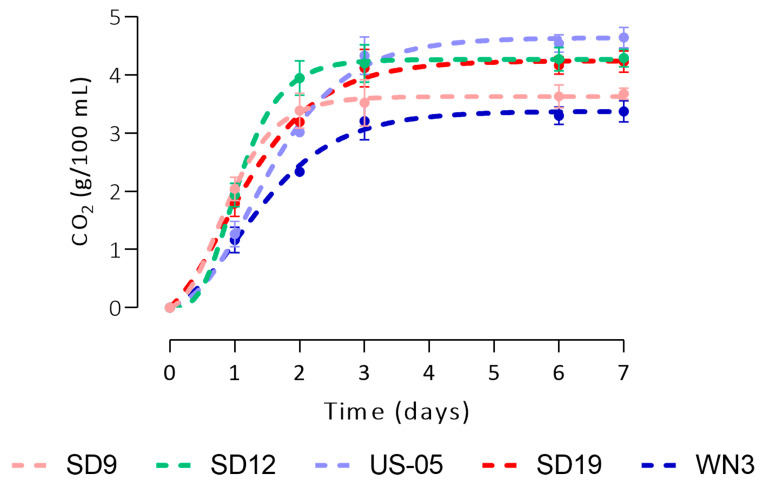
Dynamics of CO_2_ production by the 5 selected *S. cerevisiae* strains, modelled according to the Gompertz equation.

**Figure 3:**
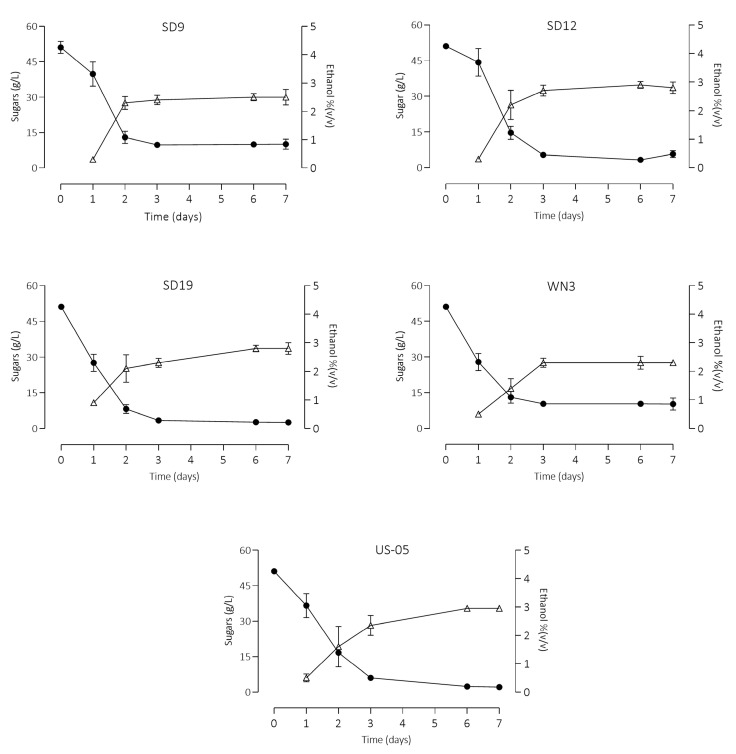
Total sugars (maltose, maltotriose, fructose, and glucose) (●) and ethanol (∆) trends (mean ± standard deviation) during the malt wort fermentations of the 5 S. cerevisiae strains (SD9, SD12, WN3, SD19, and US-05), inoculated at 2 × 10^6^ CFU/mL.

The selected strains were tested for their fermentation activity in wort in order to select the best performing for small-scale fruit and control beer production. Yeast initial concentration was 2 × 10^6^ CFU/mL. Fermentations were carried out in duplicate at 20 °C and monitored for seven days by assessing CO_2_ evolution determined by the flask weight loss (Figure 2), total sugars and ethanol (Figure 3).

Starting from the third day, the amount of CO_2_ became stable in all the trials, with *S. cerevisiae* WN3 showing the worst performance. The CO_2_ production was modelled according to the Gompertz equation, obtaining the kinetic parameters C (maximum yield), µ max (maximum rate), and Lag (length of the lag phase) (Supplementary Table S1). The goodness of fit of this model was appropriate for all the tested strains, with R^2^ values higher than 0.99. WN3 strain confirmed the worst performances in terms of maximum yield and µ max, whereas *S. cerevisiae* SD19 and US-05 showed the highest µ max and yields. Concerning sugar consumption (Figure 3), after the first day, the percentage of sugar reduction ranged from 13% (SD12) to 46% (SD19); after the second day, the highest concentrations were observed in the samples WN3 and US-05, ca. 16 g/L final concentration. After the third day, the sugar content was above 9 g/L only in the samples inoculated by the strains SD9 and WN3, independently of the type of inoculum. Ethanol production reflected sugar consumption trends, with the production peaks that differed between high and low inoculum.

Table 2 shows the chemical and microbiological composition of the fermented worts after seven days of fermentation.

The samples characterised by the highest residual sugars were those inoculated by strains SD9 and WN3. The highest residual sugar content was derived from the scarce maltotriose utilisation. Indeed, a reduction of less than 5% of this sugar was observed. Results confirmed that the utilization degree, as well as the ability to utilise these sugars, is strain dependent [27]. Glucose and fructose were used as the primary carbon sources despite the higher initial concentration of maltose and maltotriose. Indeed, monosaccharides are the preferred carbon source for *S. cerevisiae*; hence, the expression of alternative sugar utilisation enzymes is repressed when glucose is present in the growth medium. Ethanol concentration ranged from 2.30% by WN3 (lowest percentage) to 2.95% reached by US-05. The lower alcohol production observed in the other fermented worts (SD9 and WN3) might be due to low maltotriose utilization, which corroborated their low consumption shown in the preliminary in vitro results. As regards glycerol, the lowest production was observed by inoculating the commercial strain. After the seven days of fermentation, cell viability was also assessed. *S. cerevisiae* SD12 exhibited the highest cell concentration, while the lowest yeast concentration was shown by *S. cerevisiae* SD9.

Based on the results, for small-scale fruit and control beer production, *S. cerevisiae* SD12 and SD19, isolated from sourdoughs, were selected; they exhibited a higher maltotriose consumption, low acetic acid production, and high viability. US-05 strain was used as a control. As reported by Gonçalves et al. [28], among various food matrices from which new brewing yeasts may be derived, baking seems to be one of the most promising. Indeed, brewing and baking yeasts share a genetic relatedness regarding the uptake and assimilation of maltose and maltotriose. Previous studies suggested that sourdough *S. cerevisiae* strains can be used as starter cultures to ferment brewer’s wort. Catallo et al. [29] highlighted the potential use of maltose and maltotriose-positive sourdough *S. cerevisiae* for sahti ale (a typical Finnish beer) production. However, Rossi et al. [30] observed a better fermentative capability of strains from grape must. However, a better volatile profile (particularly esters production) of a sourdough strain in the wort. Since alcohol content development was low for a beer (2.5–3.0% *v/v* compared to the standard of ca 5% *v/v*), the wort was prepared by using more commercial preparation in order to increase the initial sugar amount.

### 3.3. Fruit and Control Beer Production

**Figure 4:**
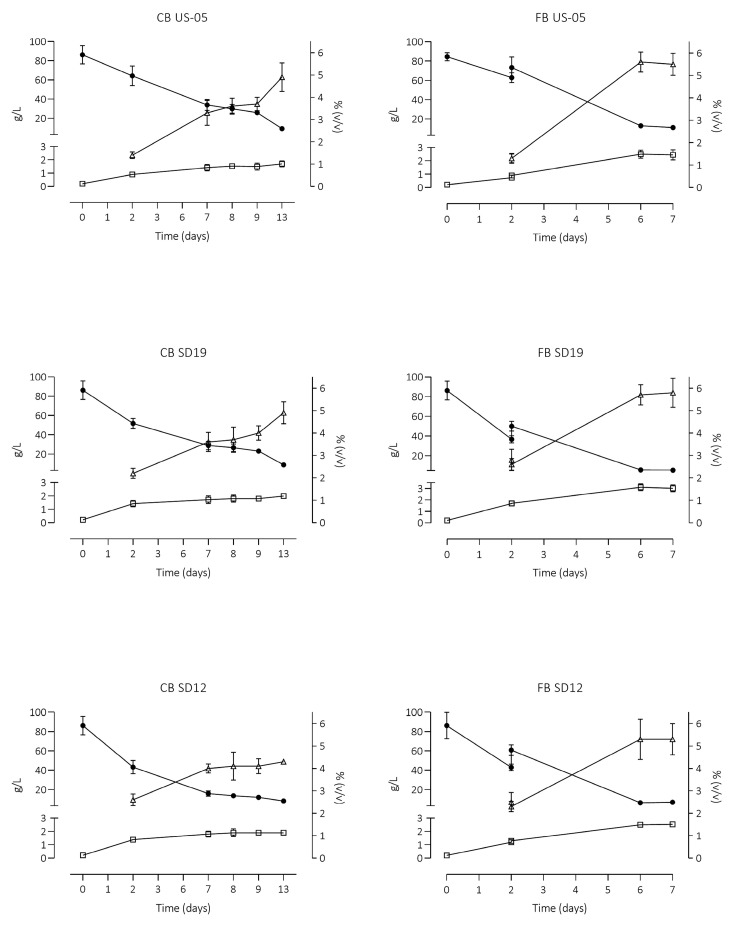
Total sugars (maltose, maltotriose, fructose, and glucose) (●), glycerol (□), and ethanol (∆) trends (mean ± standard deviation) during the primary fermentations of the 3 selected strains of *S. cerevisiae* (US-05, SD12, and SD19), inoculated for fruit beer (FB) and control beers (CB) production. Total sugars and glycerol are plotted on the left axis; ethanol is plotted on the right axis.

The selected strains, SD12, SD19, and US-05, were used to carry out the wort primary fermentation in a volume of 5 L. The raspberry puree was also analysed, and it was composed of glucose 91 g/L, fructose 88 g/L, and characterised by a pH of 3.13. Figure 4 displays the sugars, glycerol and ethanol evolution during primary fermentation.

The fermentations were considered to end when the sugar concentration was around 10 g/L. After the second day of primary fermentation, the raspberry puree was added in 15% (150 g/L), a percentage commonly used in fruit beer production, for fruit beer production. Interestingly, sugar depletion occurred at different times depending on the raspberry puree addition. The puree addition provided additional sugars, particularly glucose and fructose (ca. 10 g/L each), which boosted yeast activity. Despite the inclusion of additional sugars, their depletion occurred earlier compared to the control beers. Indeed, regardless of the inoculated strains, the sugar consumption was faster in beer integrated with raspberry puree (complete depletion within seven days, compared to thirteen days without addition).

As reported in Figure 4, sugar reduction after two days was lower in wort inoculated by US-05, ca 25%, and total sugars were still more than 60 g/L. However, with puree addition, a content of less than 10 g/L of total sugars was reached after seven days, while in the control, it occurred after thirteen days of fermentation. Our results are consistent with those of Siesto et al. [31], who observed a higher consumption of fermentable sugars and CO_2_ production by three autochthonous *S. cerevisiae* with the addition of 25% of grape must.

As regards yeast metabolites, raspberry puree addition affected the glycerol content, which was above 2.45 g/L (3.01 in the SD19 strains) after seven days of primary fermentation, whereas, in the control beers, glycerol content did not exceed 2.0 g/L. The same boosting effect of the puree was observed for ethanol production, which was more pronounced in the fruit beer samples, above 5% (*v*/*v*). This was probably due to the supplementary sugars provided by puree and, to some extent, to vitamins and minerals required for yeast growth present in fruit puree [2]. Together with chemical analyses, microbiological analyses were performed on the six samples at the end of the primary fermentation, which occurred on the 7th and 13th days for control beers and fruit beers, respectively. Final cell concentrations of yeasts indicated higher viability in the samples with raspberry compared to the control beers when inoculated by the SD12 and US-05 strains, (3.38 ± 0.31) × 10^7^ CFU/mL and (3.23 ± 0.25) × 10^7^ CFU/mL in comparison to (1.03 ± 0.11) × 10^7^ CFU/mL and (1.31 ± 0.21) × 10^7^ CFU/mL. SD 19 strain did not exhibit different viability between the beers, (1.17 ± 0.30) × 10^7^ CFU/mL and (1.40 ± 0.12) × 10^7^ CFU/mL, in CB and FB beer, respectively. The addition of raspberry, as expected, affected the pH by lowering the values below 4.0. After the primary fermentation, beers were refermented in bottles for 15 days at 20 °C, adding sucrose (5 g/L) and allowing the carbonation of the beers.

### 3.4. Physico-Chemical Characteristics of Control (CB) and Fruit (FB) Beers

The physicochemical features of the beers after the refermentation period in bottles are reported in Table 3. The data were subjected to a two-way analysis of variance (Supplementary Table S2) in order to evaluate the effect of the inoculated strain, the addition of raspberry puree, and their interaction with the beer characteristics.

Sugar concentrations were affected by the interaction of strain and raspberry addition. Indeed, the fruit beers displayed a higher residual sugar content with some differences depending on the inoculated strain. The fruit beer inoculated with US-05 showed a higher sugar content compared to the others and was not different from the control beers. Maltose concentration was below 2 g/L in each sample, with an average reduction from the initial concentration of 97.5%, whereas the maltotriose decrement was lower, with an average value of 76.5%. Glucose and fructose, as in the previous trials, were almost depleted. The glycerol amount was influenced by both the strain and raspberry addition. Fruit beers showed overall a higher concentration of this compound, more than 2.5 g/L, compared to control beers, in which it did not exceed 2 g/L. The sample inoculated by SD19 was characterised by the highest glycerol content among fruit beers. Glycerol is the third largest fermentation product after ethanol and carbon dioxide; its biosynthesis is predominantly strain-dependent [32]. It has an influence on the sensory traits of fermented beverages, contributing to body and mouthfeel. Similarly to Siesto et al. [31], the highest glycerol amount was found in samples with the highest ethanol content, even if we observed lower values compared to them, up to 7.15 g/L and 3.06 g/L, respectively. Glycerol is produced by *S. cerevisiae* in response to the stressful conditions caused by high ethanol content in the environment [33]. As regards ethanol, the only statistically significant parameter was the raspberry addition. Accordingly, the fruit beers showed a generally higher alcohol content (above 5.3%) even if with some differences and in the suitable range for this type of product. Yin et al. [10] found a slightly higher ethanol content in red raspberries beer of 5.7–5.8%. CB SD12 sample was characterised by the lowest percentage. The beer’s pH was strongly related to the raspberry addition and the strain. Concerning the raspberry addition, the decrement in the pH was mostly related to the pH of puree (3.13), lower than that of malt, which led to a similar pH in the fruit beers, all below 4.0. Some differences based on the strain were observed in the control beers, where US-05 showed the lowest pH, 4.12. The fruit beer’s pH is consistent with those reported by Nardini and Garaguso [34], which determined pH of 3.76 and 3.64 in a Lambic and Ale beer added with raspberry, respectively. Some differences based on the strain observed in the control beers might be related to certain organic acids production by *S. cerevisiae*. Although raspberries are a well-known source of phenolic compounds, our data did not suggest an effect on the total phenolic compounds (TPC) of beer determined by the Folin–Ciocalteu assay. TPC values ranged from 700.30 ± 69.0 mg GAE/L to 743.82 ± 62.60 mg GAE/L in CB and from 748.07 ± 60.36 mg GAE/L to 824.05 ± 36.73 mg GAE/L in FB. In comparison to other fruit beers, these concentrations are higher; indeed, Nardini and Garaguso [34] found values ranging from 465 to 536 mg/L in ale beer and lambic beer with raspberry; Ducruet et al. [9] reported 623 mg GAE/L in the goji berries beer; Martinez et al. [35] detected TPC content ranging from 555.23 ± 27.00 of the control beer to 290.34 ± 9.63 mg GAE/L of the beer with the highest amount of persimmon; Yin et al. [10] determined a TPC of 696 mg GAE/L in fruit beer added with red raspberries. As regards FAN amount, the observed data resulted from the interaction of raspberry and microbial starter. Fruit beers showed a final lower amount of FAN compared to the control beers; the concentrations were not statistically different among the FB sample, −93% of FAN on average.

In the control beers, the lowest FAN amount was exhibited by the commercial strain US-05, which was not different from the SD19, whereas SD12 displayed a lower FAN consumption. FAN can be defined as the sum of the individual wort amino acids, ammonium ions, and low molecular weight peptides. It is a general measure of yeast nutrients that is also used for the biosynthesis of several fermentation by-products that affect the flavour and stability of beer, such as higher alcohols, carbonyls, and esters [36,37]. The initial FAN of the wort was 281 ± 3.00 mg/L, similar to that found by Canonico et al. [20] of 268 mg/L. FAN content after refermentation is lower than the values observed by Rossi et al. [30], which ranged from 41 to 108 mg/L, and Canonico et al. [20], who pointed out a variability of results, ranging from 116 mg/L to 213 mg/L. In their work, Baigts-Allende et al. [38] highlighted a great variability among the free amino acids of commercial fruit beer, ranging from 49 to 3.9 mg/L.

### 3.5. Volatile Organic Compounds (VOCs) Analysis

**Figure 5:**
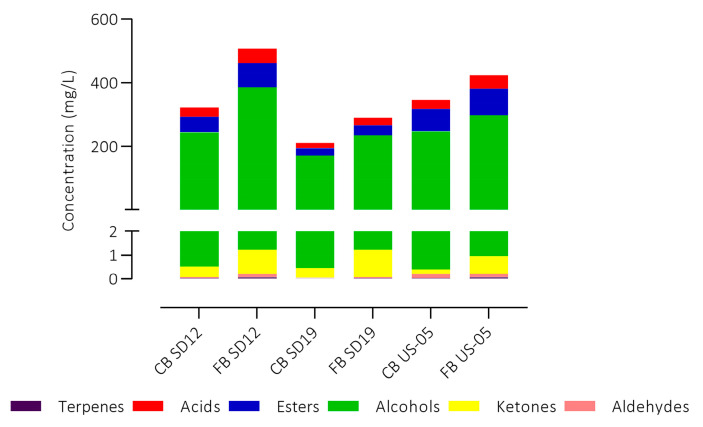
Volatile compounds concentrations expressed as the total for each chemical class in all the experimental beers.

The aromatic profiles of beers were also assessed by HS-SPME-GC/MS by detecting thirty compounds (Supplementary Table S3). Figure 5 shows the concentrations of volatile compounds expressed as total for each chemical class in the six experimental beers.

The production of aroma compounds is an industrially relevant trait for a microbial starter; indeed, the majority of flavour-active compounds in beer are produced during the fermentation phase and derive from yeast metabolism [39]. The highest level of aromatic compounds was found in FB SD12 beer (507.33 mg/L), followed by FB US-05 (423.90 mg/L) and CB US-05 (346.53 mg/L) and the lowest level was detected in CB SD19 (211.34 mg/L). All fruit beers contained a greater amount of aromatic compounds compared to the respective control beers, especially in terms of higher alcohol and ester content. This was probably due to a greater degree of fermentation observed in these beers and to the fruit addition, as also previously highlighted by alcohol and glycerol content. The most abundant chemical classes were represented by alcohols, followed by esters and acids in all the samples. Higher alcohols, also known as fusel alcohol, are produced by yeast during fermentation via the catabolic (Ehrlich) and the anabolic (amino acid metabolism) pathways. They are important aroma components, contributing to beer flavour by intensifying alcoholic perception and imparting a warm mouthfeel [36,39]. The highest concentration of alcohol was detected in the FB SD12 sample, followed by FB US-05, 385 mg/L and 297 mg/L, respectively. These concentrations were 36% and 16% higher compared to the respective control beers. In these samples, the alcohol amount was not statistically different, 243 mg/L and 247 mg/L, CB SD12 and CB US-05, respectively, whereas the CB SD19 exhibited a lower concentration, 171 mg/L. The most abundant alcohol detected in all the beers was 3-methyl-1-butanol, in agreement with the results shown by other authors [30,40]. Esters ranged second as a class of compounds. FB SD12 esters content was 34% higher than CB SD12, 75.02 mg/L and 49.64 mg/L, respectively, while the increase in esters of FB US-05 was 15% (82.73 mg/L FB and 69.96 mg/L in CB). Many variables are known to affect ester production, including the used yeast strain, the composition of the fermentation medium, and the production extent of alcohols [41]. Esters are produced by yeast cells during the fermentation process, largely as the result of the condensation of CoA esters of fatty acids with alcohols catalysed by intracellular enzymes. These compounds are characterised by their fruity–flowery aromas in beer [36]. As reported by several authors, among the most important esters found in beer are ethyl acetate, isoamyl acetate (responsible for banana and pear aroma), phenethyl acetate (roses, honey, sweet aroma), isobutyl acetate (fruity aroma), ethyl hexanoate (sweet apple aroma), and ethyl octanoate (sour apple aroma). Apart from isobutyl acetate, the other esters were detected in the experimental beers, with some differences in concentrations based on the fruit addition and the inoculated strains. Ethyl acetate was found in higher concentration in all the beers; these results are consistent with Olaniran et al. [42], that indicated this compound as typically present in the greatest concentration in beers. Ethyl acetate shows high importance as an aromatic constituent, conferring a fruity, solvent-like aroma [39,43]. Its amount varied among samples: SD19 showed the lowest amount, 12.52 mg/L and 13.85 mg/L, in CB and FB, respectively, whereas in FB SD19 and FB US-05, it exceeded 25–30 mg/L which is the threshold level [39]. Isoamyl acetate (responsible for banana and pear aroma) was detected in concentrations higher than its threshold of 0.6–1.6 mg/L in the beer produced by SD12 and US-05 strains, especially in beer with raspberry addition, whereas it was not detected in samples prepared with SD19 strain. Phenylethyl acetate was also detected in concentrations higher than its threshold (0.2–3.8 mg/L) in all the beers, with some differences; SD12 and US-05 beers were characterised by the highest concentrations, and as for isoamyl acetate, fruit beer showed greater content. As volatile acids, only hexanoic and octanoic acids were identified. Their total amounts ranged from 16 mg/L (CB SD19) to 45 mg/L (FB SD12); likewise, alcohols, esters, and acids content was higher in the fruit beers with respect to the control beers. Diacetyl was found in a concentration above the threshold level (0.9 mg/L) in all the experimental beers. It is vicinal diketone formed during beer fermentation as a by-product of amino acid synthesis, responsible for the butter- or butterscotch-like flavour [44]. Although it is generally considered an off flavour and unwanted in beers, the sensory perception of diacetyl is highly dependent on the beer matrix and style, being particularly detectable in lager beers [45]. Among aldehydes in beer, acetaldehyde is recognised as the most important one, occurring as an intermediate product in alcoholic fermentation. It is responsible for green apple flavour, and it is usually higher at the beginning of the fermentation. The higher concentrations were found in the SD12 and US-05 samples.

**Figure 6:**
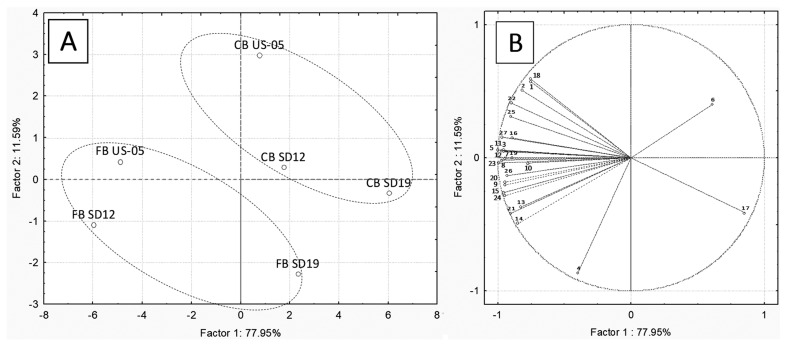
Principal component analysis (PCA). Score plot (**A**): projection of the samples on the factor plane. Loading plot (**B**): projection of the variables on the factor plane. Fruit beer (FB) and control beer (CB) obtained using three S. cerevisiae strains, SD12, SD19 and US-05. Variables: (1) acetaldehyde, (2) ethyl acetate, (3) ethyl isobutyrate, (4) diacetyl, (5) 1-propanol, (6) 2-methyl-1-propanol, (7) isoamyl acetate, (8) 1-butanol, (9) 3-methyl-1-butanol, (10) ethyl hexanoate, (11) hexyl acetate, (12) p-cymen, (13) Z3 hexenyl acetate, (14) ethyl lactate, (15) 1-hexanol, (16) ethyl octanoate, (17) benzaldehyde, (18) ethyl decanoate, (19) diethyl succinate, (20) α terpineol, (21) ethyl benzenacetate, (22) phenylethyl acetate, (23) hexanoic acid, (24) β-damascenone, (25) ethyl dodecanoate, (26) phenylethyl alcohol, and (27) octanoic acid.

In order to assess the volatile compounds that were significantly affected by the inoculated yeast strain, the addition of raspberry puree, and their interaction, a two-way ANOVA was carried out (Supplementary Table S3). Based on the ANOVA results, among the thirty detected compounds, 4-ethylguaiacol, 4-ethylphenol and benzyl alcohol were not included in the principal component analysis (PCA). Figure 6A shows the distribution of the samples in the first two principal components for the considered variables, while in Figure 6B, the score plot, indicating the influence of the variables in the factor plane, is shown.

The first component (PC1) explains approximately 78% of the total variance, whereas the second component (PC2) is 12%. The PCA corroborated the effect of the raspberry addition and of the strains on beer characteristics. All the control samples are located upper on the right side of the plane compared to the respective fruit beers. As reported in Figure 6B, 2-methyl-1-propanol is positively linked to the control beers, where it is found at higher concentrations compared to fruit beers. This compound is responsible for the alcohol aroma, even if, in all the samples, it did not exceed the threshold value of 65 mg/L [46]. On the opposite, ethyl-lactate, 1-hexanol and β-damascenone were negatively linked to control beers, being found in higher concentrations in the fruit beers. The inoculated strain also affected the aroma profile of beers, leading to a distribution on the plane mostly correlated with PC1. As a result, the beers obtained by the US-05 and the sourdough strain SD12 share more similarities compared to the beers obtained by the SD19 strain (Figure 6A) and were richer in aroma compounds, both in control and fruit beers. Notably, ethyl acetate, ethyl decanoate (fruity aroma), and phenylethyl acetate were above their threshold values. Benzaldehyde concentration, responsible for almond, burnt sugar flavour, was greater in the SD19 samples.

## 4. Conclusions

Recent interest in the special beer category has encouraged the search for novel brewing materials, including new ingredients and novel yeast strains, in order to differentiate the flavour and aroma characteristics of the finished products. In this study, a selection of several brewing characteristics of *S. cerevisiae* strains isolated from sourdoughs and wineries was carried out. Strain fermentation properties were also assessed on wort, thus allowing the choice of two sourdough *S. cerevisiae* strains, able to utilise maltose and maltotriose, which were used for fruit beer production with raspberry puree addition and control beers without fruit. Two-way ANOVA indicated the effect of both microbial strain and raspberry addition on the chemical features of the produced beers. Fruit beers were characterised by higher ethanol and glycerol production due to an enhanced yeast activity determined by fruit inclusion and by a lower pH compared to control beers. Raspberry addition also affected the VOCs profile of beers, leading to a significant enrichment in higher alcohols and esters with some differences based on the inoculated strain. Thus, this work showed the suitable application of a non-brewing *S. cerevisiae* strain for beer production that, together with fruit addition, can be exploited for obtaining a beer with peculiar and typical features. Nevertheless, this study showed that yeast strains, isolated from different food sources, represent a reservoir of novel starter cultures that can contribute to beer differentiation, particularly desired in the craft beers sector. Further studies will be necessary to test the application of the strains for a larger scale beer production and to assess the sensory and nutritional properties of the fruit beers.

## Figures and Tables

**Table 1:** Strains of *S. cerevisiae* used in the study and their source of isolation.

Source of Isolation	Strain
Sourdough	SD1; SD2; SD3; SD4; SD5; SD6; SD7; SD8; SD9; SD10; SD11; SD12; SD13; SD14; SD15; SD16; SD17; SD18; SD19
Winery	WN1; WN2; WN3
Commercial bakery yeast	ZEUS 45DB3 (Zeus Iba s.r.l, Florence, Italy)
Commercial brewing yeasts	Fermentis SafAle US-05 (Fermentis, LeSaffre, Italia S.p.A., Parma, Italy); FERMOALE AY3 (AEB group, San Polo (BS) Italy); Weiss arome+ (AEB group, San Polo (BS) Italy); Fermentis SafAle F2 (Fermentis, LeSaffre, Italia S.p.A., Parma, Italy)
Commercial wine yeasts	Lalvin S6U (Lallemand Inc., Montreal, QC, Canada), Lalvin EC1118 (Lallemand Inc., Montreal, QC, Canada)

**Table 2:** Chemical and microbiological composition of 7 day-fermented worts obtained by inoculating the 5 *S. cerevisiae* strains at 2 × 10^6^ CFU/mL. Total sugars: sum of maltotriose, maltose, glucose, and fructose content.

*S. cerevisiae* Strain	Maltotriose (g/L)	Maltose (g/L)	Glucose (g/L)	Fructose (g/L)	Total Sugars (g/L)	Glycerol (g/L)	Acetic Acid (g/L)	Ethanol (%*v/v*)	Yeast Concentration (CFU/mL)
US-05	1.35 ± 0.07 ^a^	0.20 ± 0.00	<0.10	0.55 ± 0.07	2.10 ± 0.00 ^a^	0.95 ± 0.07 ^a^	<0.01 ^a^	2.95 ± 0.07 ^c^	(2.81 ± 0.70) × 10^7 a^
SD9	9.20 ± 0.14 ^d^	0.50 ± 0.00	<0.10	0.40 ± 0.14	10.05 ± 0.07 ^b^	1.15 ± 0.07 ^b^	0.20 ± 0.10 ^c^	2.45 ± 0.20 ^ab^	(9.25 ± 0.79) × 10^6 a^
WN3	9.00 ± 0.00 ^d^	0.95 ± 0.07	<0.10	0.50 ± 0.1	10.25 ± 0.07 ^b^	1.05 ± 0.07 ^ab^	0.14 ± 0.03 ^bc^	2.30 ± 0.21 ^a^	(5.14 ± 0.48) × 10^7 ab^
SD19	2.05 ± 0.07 ^b^	0.30 ± 0.01	<0.10	0.30 ± 0.04	2.65 ± 0.07 ^a^	1.10 ± 0.00 ^ab^	0.08 ± 0.02 ^b^	2.80 ± 0.25 ^bc^	(4.45 ± 0.14) × 10^7 ab^
SD12	5.10 ± 0.00 ^c^	0.40 ± 0.00	<0.10	0.50 ± 0.05	5.80 ± 0.00 ^ab^	1.05 ± 0.07 ^ab^	0.08 ± 0.02 ^b^	2.80 ± 0.14 ^bc^	(6.98 ± 2.01) × 10^7 c^

Values are expressed as mean ± standard deviation. Values of the same column with different letters are significantly different (*p* < 0.05).

**Table 3:** Physico-chemical features (mean ± standard deviation) of fruit beers and control beers (without puree addition) after 15 days of bottle refermentation. FB—Fruit beers with raspberry addition. CB—control beers without fruit addition. Total sugars—sum of maltotriose, maltose, fructose, and glucose amount. Different letters in the same column indicate statistically significant differences (*p* < 0.05).

Sample	Maltotriose (g/L)	Maltose (g/L)	Glucose (g/L)	Fructose (g/L)	Total Sugars (g/L)	Glycerol (g/L)	Ethanol (%*v*/*v*)	pH	Total Phenolic Content (mg GAE/L)	FAN (mg/L)
FB US-05	4.15 ± 0.06 ^bc^	1.20 ± 0.07 ^abc^	<0.10	0.81 ±0.05 ^b^	6.27 ± 0.04 ^c^	2.53 ± 0.05 ^b^	5.75 ± 0.21 ^bc^	3.55 ± 0.05 ^a^	748.07 ± 60.36 ^a^	14.65 ± 1.61 ^a^
FB SD19	3.45 ± 0.07 ^a^	1.15 ± 0.07 ^ab^	<0.10	0.42 ± 0.03 ^a^	5.10 ± 0.10 ^a^	3.06 ± 0.07 ^c^	6.05 ± 0.63 ^c^	3.69 ± 0.10 ^a^	824.05 ± 36.73 ^a^	17.78 ± 1.42 ^a^
FB SD12	3.80 ± 0.01 ^ab^	0.98 ± 0.32 ^a^	<0.10	0.42 ± 0.03 ^a^	5.27 ± 0.38 ^ab^	2.60 ± 0.07 ^b^	5.40 ± 0.15 ^abc^	3.78 ± 0.05 ^a^	767.02 ± 62.01 ^a^	21.02 ± 1.47 ^a^
CB US-05	4.00 ± 0.14 ^bc^	1.65 ± 0.20 ^bc^	<0.10	0.58 ± 0.08 ^a^	6.28 ± 0.20 ^c^	1.76 ± 0.15 ^a^	4.80 ± 0.30 ^abc^	4.12 ± 0.10 ^b^	743.82 ± 62.60 ^a^	24.00 ± 2.64 ^ab^
CB SD19	4.25 ± 0.21 ^c^	1.25 ± 0.07 ^abc^	<0.10	0.41 ± 0.01 ^a^	5.97 ± 0.30 ^bc^	1.92 ± 0.18 ^a^	4.60 ± 0.42 ^ab^	4.45 ± 0.05 ^bc^	700.30 ± 69.0 ^a^	31.54 ± 2.52 ^b^
CB SD12	4.13 ± 0.04 ^bc^	1.85 ± 0.07 ^c^	<0.10	0.43 ± 0.03 ^a^	6.52 ± 0.05 ^c^	1.87 ± 0.09 ^a^	4.05 ± 0.07 ^a^	4.58 ± 0.10 ^c^	716.61 ± 43.18 ^a^	64.60 ± 4.52 ^c^

## Data Availability

Data are contained within the article or Supplementary Material.

## Supplementary Materials

The following supporting information can be downloaded at: https://www.mdpi.com/article/10.3390/foods12183354/s1, Figure S1: Growth of the tested *S. cerevisiae* strains on YEPD with maltose at 12 °C, YEPD with maltose at 20 °C and YEPD with maltotriose, determined by measuring the absorbance (OD) after 48 h; Table S1: Kinetic parameters of CO_2_ production during 7 days of fermentation in malt wort by the 5 *S. cerevisiae* strains inoculated at 2 × 10^6^ CFU/mL; Table S2: Results of two-way ANOVA for the considered parameters (inoculated *S. cerevisiae* strain, raspberry addition, and their interaction) on beer characteristics. Ns: not significant (*p* < 0.05); Table S3: Volatile compounds (mean ± std.dev) detected by HS-SPME-GC/MS of the experimental beers inoculated by three *S. cerevisiae* strains (SD12, SD19, and US-05). CB: control beers; FB: fruit beers with raspberry addition. Two-way ANOVA using as a factor the yeast strain, the addition of raspberry and their interaction.
