# Supplementary material for: Exploitation of Selected Sourdough Saccharomyces cerevisiae Strains for the Production of a Craft Raspberry Fruit Beer
Source: Foods. 2023 Sep 7;12(18):3354. doi: 10.3390/foods12183354 (PMC10529207; doi:10.3390/foods12183354)
Supplement: Supplementary file 1 [file foods-12-03354-s001.zip › Table S1.pdf]

Table S1. Kinetic parameters of CO_2 production during 7 days of fermentation in malt wort by the 5 *S. cerevisiae* strains inoculated at $2x10^6$ CFU/mL.

**Kinetic parameters**				
Strain	µ max (dCO_2/100 mL h^{-1})	Lag (h)	C (CO_2/100mL)	R^2
US-05	2.35±0.20[b]	0.80±0.10	4.41±0.15[b]	0.99
SD9	2.89 ± 0.11[b]	0.83 ± 0.02	3.44 ± 0.04[a]	0.99
WN3	1.57±0.21[a]	0.64±0.18	3.10±0.18[a]	0.99
SD19	2.40±0.13[b]	0.58±0.06	4.09±0.09[b]	0.99
SD12	2.89±0.10[b]	0.83±0.02	3.44±0.04[a]	0.99

Values in the same column with different letters (a–d) are significantly different ($p<0.05$). The data are the means of three independent experiments ± standard deviations.
