# Supplementary material for: Exploitation of Selected Sourdough Saccharomyces cerevisiae Strains for the Production of a Craft Raspberry Fruit Beer
Source: Foods. 2023 Sep 7;12(18):3354. doi: 10.3390/foods12183354 (PMC10529207; doi:10.3390/foods12183354)
Supplement: Supplementary file 1 [file foods-12-03354-s001.zip › Table S2.pdf]

Table S2. Results of two-way ANOVA for the considered parameters (inoculated *S. cerevisiae* strain, raspberry addition, and their interaction) on beer characteristics. Ns: not significant (p<0.05).

	**Strain**	**Raspberry addition**	**Interaction**
	p-value	p-value	p-value
Maltotriose	ns	0.0022	0.0028
Maltose	ns	0.0026	0.0461
Fructose	0.0002	0.0233	0.0195
Total sugars	0.0088	0.0013	0.0181
Glycerol	0.012	<0.0001	ns
Ethanol	ns	0.0008	ns
pH	0.002	<0.0001	ns
Total phenolic content (mg GAE/L)	ns	ns	ns
FAN (mg/L)	<0.0001	<0.0001	0.0002
