# Supplementary material for: Exploitation of Selected Sourdough Saccharomyces cerevisiae Strains for the Production of a Craft Raspberry Fruit Beer
Source: Foods. 2023 Sep 7;12(18):3354. doi: 10.3390/foods12183354 (PMC10529207; doi:10.3390/foods12183354)
Supplement: Supplementary file 1 [file foods-12-03354-s001.zip › Table S3.pdf]

Table S3. Volatile compounds (mean±std.dev) detected by HS-SPME-GC/MS of the experimental beers inoculated by three *S. cerevisiae* strains (SD12, SD19, and US-05). CB: control beers; FB: fruit beers with raspberry addition. Two-way ANOVA using as factor the yeast strain, the addition of raspberry and their interaction.

Classes	Compounds (mg/L)	CB SD12	FB SD12	CB SD19	FB SD19	CB US-05	FB US-05	Two-way ANOVA
Aldehydes-Ketones	Acetaldehyde	0.04±0.00	0.15±0.01	0.02±0.00	0.04±0.00	0.18±0.01	0.17±0.01	*p* strain < 0.0001<br>*p* raspberry 0.0001<br>*p* interaction < 0.0001
	Benzaldehyde	0.001±0.000	0.000±0.000	0.001±0.000	0.001±0.000	0.000±0.000	0.000±0.000	*p* strain < 0.0001<br>*p* raspberry 0.0005<br>*p* interaction 0.0027
	Diacetyl	0.44±0.01	1.00±0.03	0.41±0.01	1.16±0.03	0.19±0.00	0.72±0.02	*p* strain < 0.0001<br>*p* raspberry <0.0001<br>*p* interaction 0.0008
Alcohols	1-Propanol	25.87±1.81	42.12±2.95	15.29±1.07	22.77±1.59	28.24±1.98	38.74±2.71	*p* strain < 0.0001<br>*p* raspberry < 0.0001<br>*p* interaction ns
	2-Methyl -1-Propanol,	21.33±1.07	9.61±0.48	18.75±0.94	5.76±0.29	15.91±0.80	3.71±0.18	*p* strain < 0.0001<br>*p* raspberry < 0.0001<br>*p* interaction ns
	1-Butanol	1.47±0.03	1.99±0.40	1.14±0.02	1.38±0.03	1.52±0.03	1.65±0.03	*p* strain < 0.0001<br>*p* raspberry < 0.0001<br>*p* interaction 0.0003
	3-Methyl-1-Butanol	142.08±9.95	242.87±17.00	97.82±6.85	145.21±11.62	140.67±11.25	185.60±14.77	*p* strain 0.006<br>*p* raspberry 0.0001<br>*p* interaction 0.0299
	1-Hexanol	0.07±0.00	0.12±0.00	0.04±0.00	0.08±0.00	0.06±0.00	0.12±0.00	*p* strain < 0.0001<br>*p* raspberry 0.0001<br>*p* interaction 0.0158
	Benzyl Alcohol	0.72±0.04	0.77±0.04	0.70±0.03	0.73±0.04	0.73±0.04	0.75±0.04	*p* strain ns<br>*p* raspberry ns<br>*p* interaction ns
	Phenylethyl Alcohol	52.44±3.15	88.11±5.29	43.04±2.53	58.10±3.47	247.63±12.39	29.83±14.90	*p* strain < 0.0009<br>*p* raspberry 0.0001<br>*p* interaction 0.0050

Esters	Hexyl acetate	0.002±0.000	0.006±0.000	0.000±0.000	0.000±0.000	0.002±0.000	0.005±0.000	*p* strain < 0.0001<br>*p* raspberry < 0.0001<br>*p* interaction < 0.0001
	Ethyl acetate	24.18±0.98	26.71±1.07	12.53±0.50	13.86±0.55	25.75±1.03	28.51±1.14	*p* strain < 0.0001<br>*p* raspberry 0.0057<br>*p* interaction ns
	Ethyl isobutyrate	0.009±0.000	0.02±0.00	0.003±0.000	0.01±0.000	0.01±0.000	0.02±0.001	*p* strain < 0.0001<br>*p* raspberry < 0.0001<br>*p* interaction < 0.0001
	Isoamyl acetate	2.42±0.20	7.79±0.62	0.000±0.000	0.000±0.000	2.22±0.18	5.95±0.48	*p* strain < 0.0001<br>*p* raspberry < 0.0001<br>*p* interaction < 0.0001
	Ethyl hexaonate	0.79±0.04	0.92±0.05	0.23±0.01	0.31±0.02	0.40±0.02	0.67±0.03	*p* strain < 0.0001<br>*p* raspberry 0.0001<br>*p* interaction < 0.0116
	Ethyl lactate	2.39±0.21	3.74±0.34	2.08±0.19	3.03±0.28	2.40±0.21	3.15±0.29	*p* strain ns<br>*p* raspberry 0.0004<br>*p* interaction ns
	Ethyl octaonate	2.18±0.11	2.45±0.12	2.04±0.10	2.31±0.12	2.41±0.12	2.70±0.35	*p* strain 0.0115<br>*p* raspberry 0.0052<br>*p* interaction ns
	Ethyl decanoate	5.73±0.06	15.43±0.16	1.83±0.02	2.95±0.03	21.01±0.22	24.18±0.35	*p* strain < 0.0001<br>*p* raspberry < 0.0001<br>*p* interaction < 0.0001
	Ethyl dodecanoate	3.38±0.13	4.27±0.17	2.56±0.10	3.21±0.13	4.06±0.16	5.05±0.20	*p* strain < 0.0001<br>*p* raspberry < 0.0001<br>*p* interaction ns
	Diethyl succinate	0.06±0.00	0.08±0.00	0.04±0.00	0.06±0.00	0.06±0.00	0.10±0.00	*p* strain < 0.0001<br>*p* raspberry < 0.0001<br>*p* interaction 0.0049
	Phenylethyl acetate	8.51±0.42	13.57±0.78	4.22±0.31	5.37±0.27	11.69±0.59	12.40±0.62	*p* strain < 0.0001<br>*p* raspberry 0.0002<br>*p* interaction 0.0015

	Ethyl benzenacetate	0.000±0.000	0.001±0.000	0.000±0.000	0.001±0.000	0.000±0.000	0.001±0.000	*p* strain < 0.0001<br>*p* raspberry < 0.0001<br>*p* interaction 0.0003
	Z-3-hexenyl acetate	0.001±0.000	0.001±0.000	0.000±0.000	0.000±0.000	0.000±0.000	0.001±0.000	*p* strain < 0.0001<br>*p* raspberry <0.0001<br>*p* interaction < 0.0001
Volatile acids	Hexanoic acid	22.40±1.34	35.31±2.12	12.50±0.85	19.76±1.18	21.38±1.29	33.86±2.03	*p* strain < 0.0001<br>*p* raspberry 0.0001<br>*p* interaction ns
	Octanoic acid	6.35±0.32	10.14±0.51	3.55±0.18	4.85±0.24	7.10±0.35	8.47±0.42	*p* strain < 0.0001<br>*p* raspberry 0.0001<br>*p* interaction 0.0040
Terpenes	α terpineol	0.003±0.000	0.02±0.00	0.000±0.000	0.003±0.000	0.002±0.000	0.02±0.001	*p* strain < 0.0001<br>*p* raspberry < 0.0001<br>*p* interaction < 0.0001
	p-Cymene	0.03±0.001	0.04±0.001	0.28±0.001	0.031±0.001	0.032±0.001	0.35±0.001	*p* strain 0.0005<br>*p* raspberry 0.0002<br>*p* interaction 0.0285
	β-damascenone	0.002±0.000	0.007±0.000	0.001±0.000	0.002±0.000	0.001±0.000	0.006±0.000	*p* strain 0.0003<br>*p* raspberry < 0.0001<br>*p* interaction 0.0003
Volatile phenols	4-Ethylguaiacol	0.02±0.001	0.02±0.001	0.02±0.001	0.02±0.001	0.02±0.001	0.02±0.001	*p* strain ns<br>*p* raspberry ns<br>*p* interaction ns
	4-Ethylphenol	0.05±0.002	0.05±0.002	0.05±0.002	0.05±0.002	0.05±0.002	0.05±0.002	*p* strain ns<br>*p* raspberry ns<br>*p* interaction ns
