# Supplementary figures and images for: Exploitation of Selected Sourdough Saccharomyces cerevisiae Strains for the Production of a Craft Raspberry Fruit Beer
Source: Foods. 2023 Sep 7;12(18):3354. doi: 10.3390/foods12183354 (PMC10529207; doi:10.3390/foods12183354)

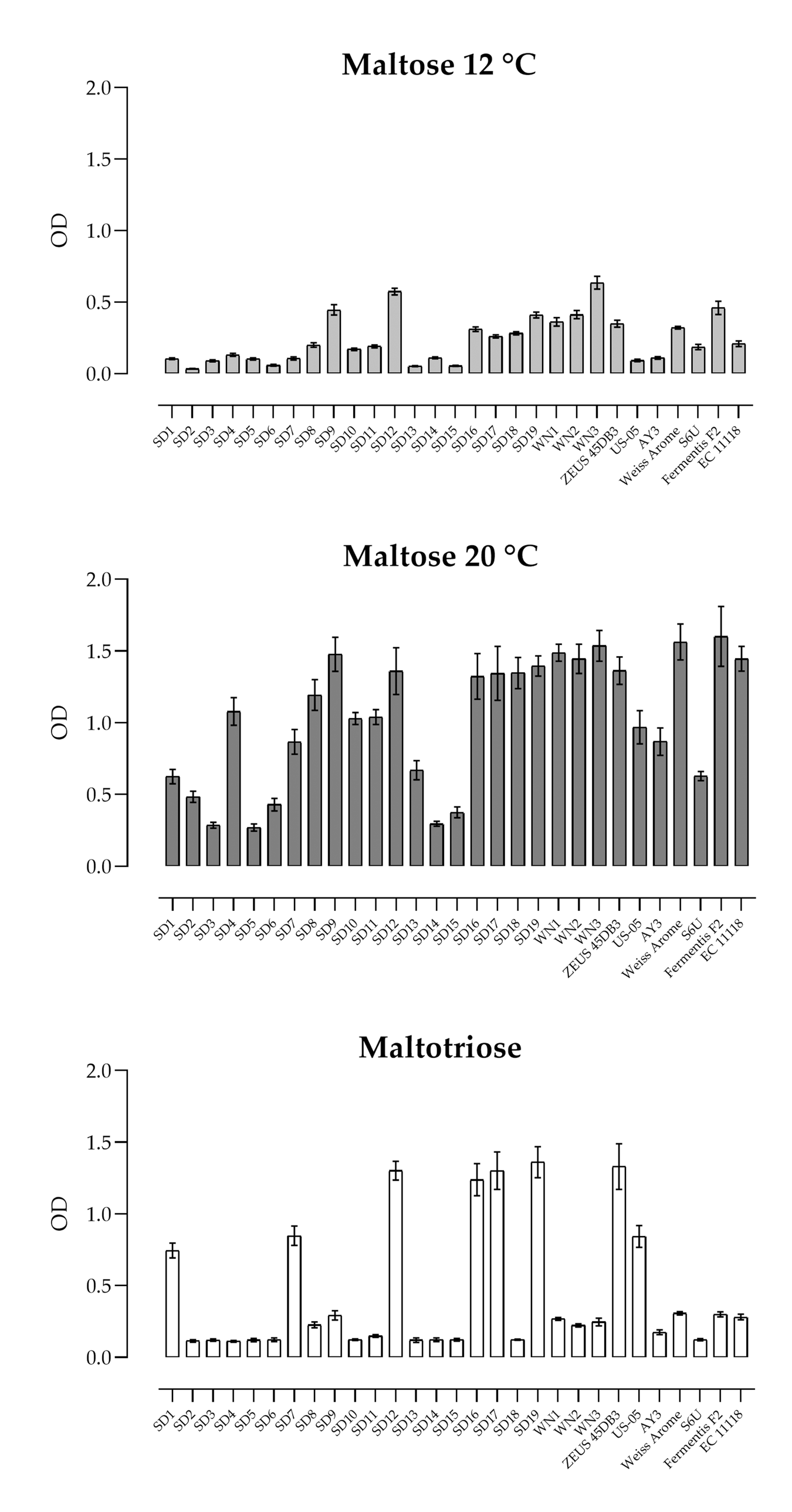

Supplement: Supplementary file 1 [file foods-12-03354-s001.zip › Fig S1.tif]
